# Supplementary material for: Improved performance of fNIRS-BCI by stacking of deep learning-derived frequency domain features
Source: PLoS One. 2025 Apr 17;20(4):e0314447. doi: 10.1371/journal.pone.0314447 (PMC12005509; doi:10.1371/journal.pone.0314447)
Supplement: Attachment — Submitted filename: Response to Reviewers.pdf [file pone.0314447.s001.pdf]

Improved Performance Of fNIRs-BCI By Stacking of Deep Learning-Derived Frequency Domain Features

Jamila Akhter[1], Hammad Nazeer[1], Noman Naseer[1 *], Rehan Naeem[1], Karam Dad Kallu[2*], Seong Young Ko[2*], Lee jiye[2]

Authors would like to extend their sincere gratitude to the editor and both reviewers for taking the time to review my manuscript. The insightful feedback and constructive suggestions are greatly appreciated and have significantly contributed to improving the quality of the work. Thank you for the valuable comments.

Reviewer 1:

Comment 1: Include a small justification for the 3cm distance between the source and the detector.

Response: Thank you for your concern. The 3 cm distance between the source and detector optodes is selected based on established literature, which identifies this spacing as optimal for measuring cortical hemodynamic responses while minimizing signal contamination from superficial layers like the scalp. This distance is widely used in fNIRS studies for effective sensitivity to cortical activity.

Comment 2: Justify why 10.1725Hz, this unusually precise rate?

Response: The 10.1725 Hz frequency is the data collection system's built-in, fixed acquisition rate, which cannot be adjusted. The hardware specifications pre-determined this precise frequency to ensure optimal performance and consistency across measurements.

Comment 3: Mention the value of 'd' and values of extinction coefficients for HbO and HbR at the used wavelength

Response: ‘d’ is the Differential path length factor, its values differ at different wavelengths. These values are hardware specifications to calculate the changes in oxy and deoxyhemoglobin concentration.

760 nm: DPF ≈ 6.2, **850 nm**: DPF ≈ 6.8

Comment 4: Include one line about the SNR response

Response: Thank you for the suggestion to add the Signal-to-Noise Ratio (SNR) in the manuscript. Manuscript has been revised to include a comprehensive explanation of SNR, detailing its significance in the context of our study. This addition clarifies how SNR is calculated and highlights its role in assessing the quality of fNIRS data.

Comment 5: Discuss data splitting strategy and overfitting response as well.

Response: Thank you for your suggestion, now the ratio of the test data (20%) and train data (80%) is added in the manuscript.

Comment 6: Revise and check for minor grammatical and English mistakes

Response: Thank you for your suggestion. Manuscript has been revised carefully to identify and correct any minor grammatical and linguistic errors to ensure clarity and readability.

Reviewer 2:

Comment 1: The abstract contains plenty of basic details and does not comprehensively address the novelty and unique outcomes of the study.

Response: Thank you for the suggestion. Following the comment, abstract has been revised which includes a comprehensive summary of the two proposed methods: stacking and FFT. Additionally, the achieved accuracies by both methods are explicitly mentioned in the abstract to provide a clear and concise overview of their performance.

Comment 2: The introduction section needs a complete rewriting as it directly begins with terminologies without leading the readers from a generalized concept to a specific one.

Response: Thank you for the suggestion. The section has been revised as follows: It begins by clearly defining BCI systems to establish their purpose and importance. Followed by a detailed explanation of the different phases involved in BCI systems. For each phase, it includes a relevant literature review to ensure comprehensive coverage of related work and to contextualize the contributions within the field.

Comment 3: The methodology section lacks attention to operational definitions and evidence. In certain cases, it simply says that literature says so, which is not an appropriate way to create an argument.

Response: Thank you for your feedback. The methodology has been reviewed and added appropriate citations for all statements derived from the literature to ensure proper attribution and strengthen the credibility of the discussion.

Comment 4: In section 2.4 there is a need to specify the version of nirsLAB utilized for preprocessing. It would be beneficial to include details about any significant features or updates in this version that might influence the preprocessing steps.

Response: Thank you for your valuable feedback. The nirsLAB version has been mentioned in the revised manuscript. This is the only version which has been utilized for the analysis.

Comment 6: In Section 2.5 it should be specified whether the features discussed are spatial or temporal, as statistical features can fall into either category. Providing this clarification will improve the precision of the analysis and help readers better understand the context of the features being utilized.

Response: Thank you for the suggestion. The nature of the features and extraction methods are discussed in detail in Section 2.5. Specifically, CNN extracts spatial features and local temporal dependencies from the time series data, while LSTM and Bi-LSTM are responsible for extracting temporal features. This clarification has been added in the revised manuscript to improve the precision of the analysis and help readers better understand the context of the features being utilized.

Comment 7: Specify the number of features extracted from each deep learning (DL) algorithm employed in the analysis. This information will enable readers to grasp the dimensionality of the processed data and understand the contribution of each algorithm to the overall feature set.

Response: Thank you for the suggestion. 32 features are extracted from each algorithm (CNN, LSTM, and Bi-LSTM), and this information is now included in the manuscript. This provides a clearer understanding of the dimensionality of the processed data and the contribution of each algorithm to the overall feature set.

Comment 8: Even for the Features extraction and channel selection section there needs to be an introductory paragraph on what they mean and imply.

Response: Thank you for your kind comment. The definition of "channels" has been discussed in in Section 2.2 and providing a detailed explanation of "feature extraction" in the dedicated feature extraction section. This clarification improves the overall understanding of these concepts in the manuscript.

Comment 9: In DL algorithms there is a need to mention the loss function, activation function, and rate of learning used for this study to extract features from the DL algorithms.

Response: According to your suggestion, the feature extraction section of the manuscript has been revised with the type of loss function and optimization algorithm, along with the learning rate used for feature extraction from the deep learning algorithms. This provides better understanding of the methodologies employed in the analysis.

Comment 10: In Figure 4 compare the filtered data with unfiltered instead of only plotting filtered data set used (HbO data set).

Response: Thank you for your suggestion, the revised figure is attached to compare filtered and unfiltered HbO concentration changes.

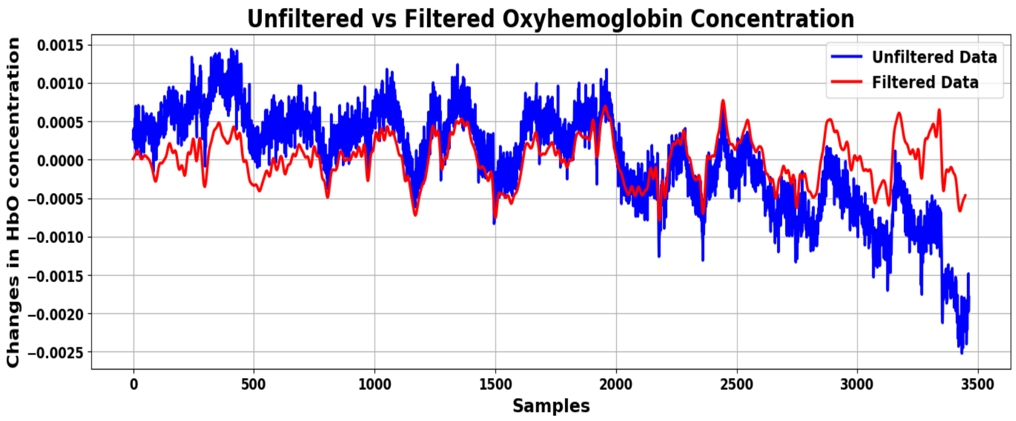

Comment 11: For the comparison of the results from the DL algorithms and proposed method it is suggested to do precision and/or F1-scores comparisons as well.

Response: Following the suggestion, a comparison of F1 scores has been added in the revised manuscript.

Comment 12: There needs to be an elaboration on what the implication would be when this methodology affects the performance based on the confusion matrix and model losses.

Response: In accordance with the suggestion, it is added in the second paragraph of the confusion matrix and losses results and F1-score results are also added to depict the improvement in precision and recall.

Comment 13: The validation section of the work has not been explained at all. It needs a thorough look with inclusion of which methodology was followed, along with evidence through citations.

Response: The database original paper has been cited in the revised manuscript. Also the results from the benchmark paper has been compared for the validation purpose with the achieved results of proposed study.

Comment 14: Lastly, while rewriting, the authors need to be elaborative on how this fft method stands unique against the previous works.

Response: fft method discussion is added in the 3rd paragraph of manuscript; mainly the comparison to other methods to improve accuracy are discussed. Thank you for the kind comment.

Comment 15: The conclusion is very simplistic. It needs to include the implication, application, limitation and future recommendations as well.

Response: Thank you for your suggestions, the application, limitations, and future recommendations are added in the conclusion section.
